# An examination of the genotyping error detection function of SIMWALK2

**DOI:** 10.1186/1471-2156-4-S1-S40

**Published:** 2003-12-31

**Authors:** Michael D Badzioch, Hawkins B DeFrance, Gail P Jarvik

**Affiliations:** 1 Department of Medicine, Division of Medical Genetics, University of Washington, Seattle, Washington, USA; 2 Division of Clinical Research, Fred Hutchinson Cancer Research Center, Seattle, Washington, USA; 3 Department of Epidemiology and Genome Sciences, University of Washington, Seattle, Washington, USA

## Abstract

This investigation was undertaken to assess the sensitivity and specificity of the genotyping error detection function of the computer program SIMWALK2. We chose to examine chromosome 22, which had 7 microsatellite markers, from a single simulated replicate (330 pedigrees with a pattern of missing genotype data similar to the Framingham families). We created genotype errors at five overall frequencies (0.0, 0.025, 0.050, 0.075, and 0.100) and applied SIMWALK2 to each of these five data sets, respectively assuming that the total error rate (specified in the program), was at each of these same five levels. In this data set, up to an assumed error rate of 10%, only 50% of the Mendelian-consistent mistypings were found under any level of true errors. And since as many as 70% of the errors detected were false-positives, blanking suspect genotypes (at any error probability) will result in a reduction of statistical power due to the concomitant blanking of correctly typed alleles. This work supports the conclusion that allowing for genotyping errors within likelihood calculations during statistical analysis may be preferable to choosing an arbitrary cut-off.

## Background

Optimal performance of genetic linkage and association tests relies on accurate and efficient genotyping as data errors reduce power to detect and map genetic effects. Even at low rates (<2%), typing errors can have significant effects on results [1]. They inflate apparent recombination and can falsely exclude linkage, especially in multipoint analysis [2]. The most insidious errors, often accounting for over 25% of all mistypings, are those not violating rules of Mendelian inheritance [1]. A recent extension to a widely used computer program, SIMWALK2 [3], has been offered to detect these errors through a Markov-chain Monte Carlo algorithm using full, extended pedigrees and multiple markers [4]. However, before application to a real data set, it is desirable to measure the program's sensitivity and specificity in detecting known genotyping errors. Low specificity or a high rate of false positives will result in undesirable loss of statistical power while low sensitivity will leave many true errors undetected. We report here a study of SIMWALK2's ability to accurately and efficiently detect known genotyping errors under a variety of conditions in a single simulated replicate of 330 pedigrees provided by the Genetic Analysis Workshop 13 organizers.

## Methods

We arbitrarily chose replicate 100 for all analysis using the initial set of simulated genotypes that were provided for those individuals who had genotypes in the Framingham Heart Study data set. The partially genotyped families, *n *= 330, contained 1701 genotyped and 2991 ungenotyped individuals and were chosen to reflect more closely a real genomic scan data set. We first determined that the marker heterozygosities (het) in the complete scan ranged from 0.582 (c17g2) to 0.919 (c2g9). We chose to analyze chromosome 22 because its seven markers encompassed this range, het = 0.607 (c22g2), 0.639 (c22g7), 0.707 (c22g3), 0.736 (c22g1), 0.762 (c22g4), 0.780 (c22g5), and 0.899 (c22g6). Two families, 232 and 309, were not genotyped for chromosome 22 markers. An average of 35.9% of the individuals were genotyped in each family. An average 6.81 markers were genotyped for each genotyped person. The complete list of typing frequencies (percentage of individuals in the family who were genotyped), numbers of families, and average family sizes are presented in Table 1.

A computer program was written to simulate genotyping errors according to the empirical error model presented by Sobel et al. [4]. For each genotype, five random numbers ranging from 0 to 1 were generated and if their values were less than or equal to a pre-set limit then a new, incorrect genotype replaced the original, correct genotype for that marker. Five data sets were created, each containing a different overall generated error rate (GER), using 0x (i.e., simulating no errors), 1x, 2x, 3x, and 4x as the pre-set (default) error rates used by SIMWALK2. SIMWALK2's five default error rates are (ε_1_) 0.0125 for false homozygosity; (ε_2_) 0.0075 for misreading one heterozygotic allele; (ε_3_) 0.005 for misreading both heterozygotic alleles; (ε_4_) 0.01 for misreading homozygote as heterozygote; and (ε_5_) 0.0025 for random mistyping (sample switch, etc.), and sum to an overall error rate of 0.0175 (ε_3 _+ ε_4 _+ ε_5_) in true homozygotes and to 0.0275 (ε_1_+ ε_2 _+ ε_3 _+ ε_5_) in true heterozygotes [4]. If more than one error type was probable for any genotype the one with the highest pre-set rate limit was simulated. This happened quite rarely and was not thought to bias the results. For each error the original and simulated genotypes were recorded as well as the error type (1 through 5). In these five data sets 0, 285, 564, 822, and 1108 genotyping errors, respectively, were generated out of a total of 11,586 chromosome 22 genotypes contained in the 330 families. Thus, the overall generated error rates were 0, 0.025, 0.049, 0.071, and 0.096. These data sets will be referred to as ge000, ge025, ge050, ge075, and ge100, respectively.

PEDCHECK [5] was used to identify all Mendelian errors in the five data sets. PEDCHECK levels 1 and 2 detect Mendelian inconsistencies between parents and children. PEDCHECK levels 3 and 4 detect occurrences of more than four alleles in full sibships and lists the relative likelihood of possible corrective measures. Errors found using PEDCHECK were untyped (reset to missing values) and the program rerun on the updated file until no more errors were found. (See Results for description of PEDCHECK-detected errors.)

MEGA2 [6] was used to prepare input files and SIMWALK2 was used to analyze the remaining genotypes in each of the five data sets, which excluded all Mendelian errors. All SIMWALK2 analyses were performed under the empiric error model. For each of the five generated data sets, five analysis error rates (AERs) were tested, 0.00001, 0.025, 0.050, 0.075, and 0.100, and are respectively referred to as ae000, ae025, ae050, ae075, and ae100. SIMWALK2's output consists of a list of identification numbers, the marker name and genotype purported to be erroneous, and the probability of mistyping of the first, second, both, and either allele(s) when any probability is greater than 0.25. The present study uses the probability that either allele was mistyped as the probability that the genotype is in error; the *p*-values given in the Results section refers to this probability.

## Results

Overall, PEDCHECK found 1104 Mendelian errors or 40% (range 38-42%) of the generated errors in the four data sets containing errors. In all but one case of 57 level 4 errors, the true misgenotyped person was listed as a possible error. Thirty-eight of these individuals were indicated as being the most likely misgenotyped person when several closely related individuals were suggested. In two cases, the individuals for whom errors were simulated were not listed in the level 4 output but others in their nuclear family were listed. Except for these latter two cases, the simulated error was untyped (even when PEDCHECK didn't indicate it was the most probable).

**Figure 1:**
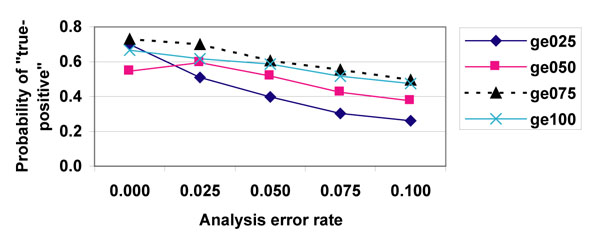
Overall probability that a purported error (*p *> 0.25) was true

When no errors were present, SIMWALK2 reported 9, 58, 124, 190, and 238 errors (*p *> 0.25) and 6, 2, 7, 7, and 5 errors (*p *> 0.95) using, respectively, AER values of 0, 0.25, 0.50, 0.75, and 0.1. Figure 1 plots "true-positive" rates and indicates that both the AER and the GER affect the probability that a purported error (*p *> 0.25) is a true (i.e., generated) error. The "false-positive" rate equals 1 minus the plotted value for any GER-AER combination. Assuming no errors (ae000) in the data sets containing errors, ratios of errors found/true errors were 7/10 (70%), 6/11 (54%), 19/26 (73%), and 12/18 (67%), respectively. At ae100, these ratios dropped to 88/337 (61%), 155/413 (37%), 260/524 (50%), and 287/604 (48%). Other than for 0.0 error rates, when the AER was equal to the GER about 50% of the purported errors were true (generated) errors.

**Figure 2:**
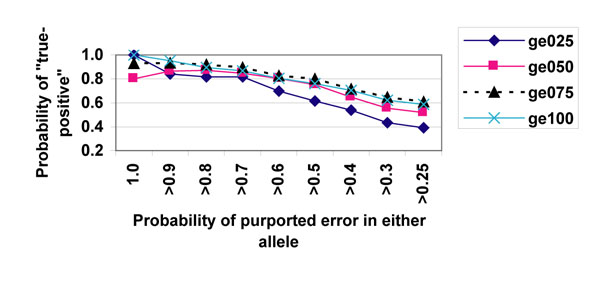
Probability of "true-positive" according to *p*-value for ae050

Expanding the ae050 results from Figure 1, Figure 2 reports the effect of increasing stringency in accepting "true-positives". Only ge100 shows a constant decline in the cumulative "true-positive" rate as more errors are accepted, from *p *= 1 (100%) to *p *> 0.25 (60%) whereas the others show at least some increase in their overall decline. Generally, however, the overall decline in the "true-positive" rate is less severe for higher GER. As the AER increases, the entire plot in Figure 2 shifts downward (data not shown).

**Figure 3:**
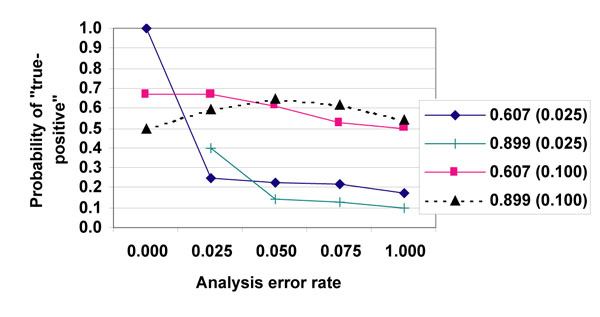
Effect of marker heterozygosity

Figure 3 examines effects of marker heterozygosity, het = 0.607 vs. 0.899, on the probability that a purported error (*p *> 0.25) was a true error that was generated under low (0.025) and high (0.100) GER. The role of GER appears to have profound effects on this probability, while marker heterozygosity does not.

**Figure 4:**
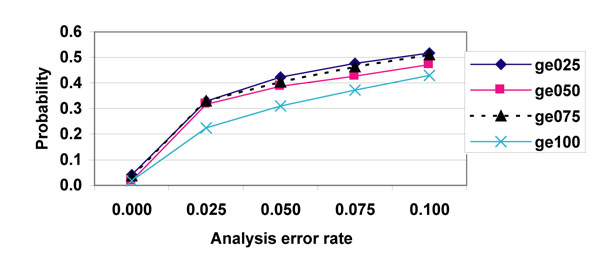
Probability that a generated error was identified (*p *> 0.25)

As a measure of success in identifying generated errors in terms of all errors present in the data set, Figure 4 indicates that increasing the AER results in an increased overall error detection rate and that the GER has less impact than AER on the overall error detection probability. It is unclear whether the lower curve for ge100 is real or due to sampling error. Over this AER range, the maximum error detection frequency was 50% (at ae100). At this highest overall rate of assumed errors, 52 % (88/170), 47% (155/328), 51% (260/509), and 43% (287/668) of the generated errors were correctly identified. However, because the relationship between AER and detection was nearly linear for AER > 0 this frequency may increase with AERs > 0.100.

**Figure 5:**
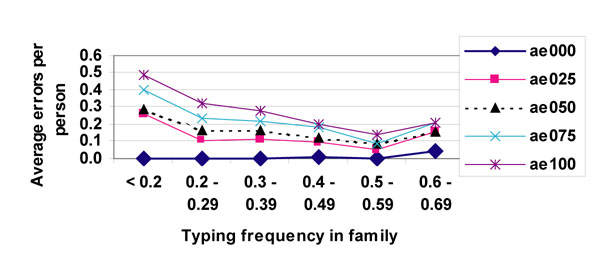
Purported error detection (*p *> 0.25) per person in ge050 according to proportion of family genotyped

Since the proportion of individuals typed in a family is also potentially a factor in identifying errors, Figure 5 presents the frequency of errors detected (*p *> 0.25) in each of six family groups defined by typing frequency. In these six groups, 35, 226, 638, 509, 230, and 63 genotyped individuals were respectively "at-risk" for a genotype error. The probability of reporting an error is generally higher in families that have a lower proportion of members genotyped. However, this trend does not appear to apply for the set of families (*n *= 9) with the highest frequency of genotyping. The small number of families and/or genotyped individuals (*n *= 63) included in this group may partially explain this apparently anomalous result.

## Conclusion

We have examined factors that potentially affect detection of Mendelian-consistent genotyping errors and SIMWALK2's ability to detect these errors under varying GERs and program-specified AERs. In this data set, up to an assumed error rate of 10% (specified in the program), only 50% of the Mendelian-consistent mistypings were found under any level of true errors. In fact, the true error rate appeared to have little impact on the proportion of errors detected. Although the chance of identifying a true error increased as the assumed error rate increased, the ratio of true positive to false-positive errors detected decreased. As many as 70% of the errors detected were false-positives. This decrease in specificity was dependent on the overall level of errors in the data and was not associated with marker heterozygosity.

Many genotyping errors will necessarily go undetected under current techniques. Under the highest assumed error rate, ae100, at least 50% of the generated errors remain undetected. These errors are consistent with Mendelian inheritance but no further examination of them was performed. Characterizing these undetected errors may provide clues leading to their identification. At present there appears to be no means by which "true-positives" can be differentiated from "false-positives" and the cost of false-positives can be quite severe. To detect true errors the investigator has no choice but to accept that this trade-off will result in loss of power in identifying genetic effects. However, our research has suggested that avenues to lower the 'overhead' costs, such as increasing the proportion of genotyped individuals per family, could be of value. Several potentially important parameters were not examined here. For instance, it is possible that allele frequency may have a significant impact on error detection rate. If a more common allele was misread as a less common one, it may be more likely to be detected as an error than otherwise. Additionally, no attempt was made to re-generate errors multiple times at a constant GER. However, the results presented here are likely robust to sampling error because most trends were smooth and consistent over varying conditions. However, any sampling variation present would be seen as differences between GER levels. Except for ae000 in Figure 1 and *p *= 1.0 in Figure 2, the plotted values were generally in proportion to GER value.

We do not attempt here to evaluate the theoretical foundation of SIMWALK2's genotyping error detection procedure but only offer a brief analysis of its function and set its results into a contextual framework. Further work in detecting true genotyping errors will no doubt be done due to its importance in linkage and association studies. Retyping suspect genotypes may help, but factors such as reproducible errors and mutations may limit its utility. Overall, we conclude that progress has been made in detecting Mendelian-consistent errors. However, blanking suspect genotypes (at any error probability) will result in a reduction of statistical power due to the concomitant blanking of correctly typed alleles. Several authors [2,7] have suggested allowing for genotyping errors within likelihood calculations during linkage analysis and this approach may be preferable to choosing an arbitrary cut-off.

## Figures and Tables

**Table 1:** Genotyping information

**Typing Frequency**	**Number of Families**	**Average Family Size**
0	2	9.0
0.08 – 0.19	18	12.5
0.20 – 0.29	72	12.7
0.30 – 0.39	117	15.6
0.40 – 0.49	72	16.3
0.50 – 0.59	40	10.8
0.60 – 0.67	9	11.3
Total (mean 0.359)	330	14.2
